# Low-intensity pulsed ultrasound inhibits adipogenic differentiation via HDAC1 signalling in rat visceral preadipocytes

**DOI:** 10.1080/21623945.2019.1643188

**Published:** 2019-07-19

**Authors:** Tianhua Xu, Kun Zhao, Xiasheng Guo, Juan Tu, Dong Zhang, Wei Sun, Xiangqing Kong

**Affiliations:** a Department of Cardiology, The First Affiliated Hospital of Nanjing Medical University, Nanjing, Jiangsu, China; b Key Laboratory of Modern Acoustics, Department of Physics, Collaborative Innovation Center of Advanced Microstructure, Nanjing University, Nanjing, Jiangsu, China

**Keywords:** LIPUS, preadipocyte, adipogenic differentiation, HDAC1, PPARγ, C/EBPs

## Abstract

Non-drug strategy targeting adipocyte differentiation is critical for alleviating visceral obesity and its related diseases. However, whether and how low intensity pulsed ultrasound (LIPUS) could be used for inhibiting visceral adipocyte differentiation is not fully understood. In this study, we aim to investigate the effect and associated mechanism of LIPUS on primary visceral preadipocyte differentiation and explore its potential role for clinical visceral obesity management. The preadipocytes were daily exposed to LIPUS (0.5 MHz, 1.2 MPa) for 10 min. Adipogenic differentiation was estimated by the formation of lipid droplets and the levels of adipogenic transcriptional factors and representative markers. Mitogen-activated protein kinase (MAPK) member proteins and histone acetylation-related molecules were measured by western blotting. LIPUS stimulation with an average acoustic pressure of 1.2 MPa led to a prominent inhibition of adipogenic differentiation and expression of adipogenic markers. As a mechanism, LIPUS treatment increased the nuclear levels of histone deacetylase 1 (HDAC1) and decreased the acetylation of histone 3 and histone 4. Meanwhile, the inhibition of the HDAC1 could block the inhibitory effect of LIPUS on adipogenic differentiation via increasing AcH3 and AcH4 levels. Our study may provide an ultrasound-based promising strategy for clinical visceral obesity control.

## Introduction

Adipose tissue, the energy storage organs, play an important role in energy homeostasis [1]. The reconstruction and increasing volume of adipose tissue are closely related to many metabolic disorder diseases, including obesity and type 2 diabetes [2,3]. Moreover, Epidemiological studies have demonstrated that visceral obesity rather than subcutaneous fat deposition plays a crucial role in cardiovascular diseases [4]. Fatty tissue is comprised of massive congregate adipocytes, and the increase in adipocyte number and size can rise the adipose tissue mass [5]. Understanding the mechanisms regulating adipocyte differentiation may provide valuable strategy for alleviating visceral obesity-related diseases.

Preadipocytes are fibroblast-like cells that are able to differentiate into fat cells in response to the appropriate induction conditions [6,7]. Adipogenic stimuli induce terminal differentiation in preadipocytes through the epigenomic activation of transcriptional factors, including peroxisome proliferator-activated receptor-γ (PPARγ) and CCAAT/enhancer binding proteins (C/EBPs) [8]. As the key transcriptional regulators of preadipocyte terminal differentiation, PPARγ and C/EBPs subsequently not only trigger positive feedback to induce their own expression, but also activate a series of downstream target genes whose expression determines the adipocyte [9]. Once the process of preadipocyte differentiation is inhibited, the mRNA and protein expression of PPARγ and C/EBPs would be down-regulated. Furthermore, many of these proteins involved in lipid metabolism and adipocyte expressed genes also will suffer a corresponding decrease and finally result in the suppression of lipid accumulation in adipocyte [1].

Histone acetylation and deacetylation could be regulated by mutual effect of histone acetyltransferases and histone deacetylases (HDACs) [10,11]. HDACs consist of four major classes and HDAC1 belongs to the class I family of HDACs, which are ubiquitously expressed and participate in acetylation of histones. In addition, HDAC1 has been implicated in a number of transcriptional processes and has been shown to interact directly and indirectly with a number of transcription factors such as p53, retinoblastoma protein, and C/EBPs [12–14]. In 2003, a study suggested that HDAC1 inhibition promoted transcriptional activation of C/EBPα and increased adipogenesis in the 3T3-L1 mouse embryonic fibroblasts model [12].

In recent years, the biological effects of ultrasound have shown significant progress in medical research. The high-intensity focused ultrasound (HIFU) technology has been applied to other areas besides cancer [15] and the thermal effect is considered the main mechanism of HIFU in the treatment of tumours [16,17]. In contrast to HIFU, LIPUS is a low-frequency and -dosage ultrasound technique that plays a dominant role in mechanical and cavitation effects. Moreover, LIPUS has been widely used in clinical treatment including bone fracture, soft tissue injury, performing thrombolytic and enhancing the therapeutic effect of drugs, etc [18–22]. In previous studies, we also found that an average intensity of 109.44 mW/cm^2^ significantly promoted rat visceral preadipocyte apoptosis via elevating P38 MAPK [23]. Whether LIPUS is also fit for the inhibition of preadipocyte differentiation is not fully understood.

**Table:** 

	HIFU	LIPUS
Common features	non-invasive, good penetrability, repeatable, focusability, obvio11lS orientation	
Dose and frequency	High	Low
Major mechanisms	thermal effect	cavitation and mechanical effect(non-thermal effect)
Major application	malignant tumors, ultrasonic lithotriJ?E	bone healing, membrane penetrability,thrombolysis
Technical requirements	high technology wntent	operation simple
\Veaknesses	high temperature scalding	lower energy

In this study, we investigated the effects of a low dose of LIPUS on visceral preadipocyte differentiation. We found that daily LIPUS stimulation with an average acoustic pressure of 1.2 MPa for 10 min significantly inhibited preadipocyte differentiation without affecting cell proliferation. Moreover, LIPUS suppressed preadipocyte differentiation via up-regulation of the HDAC1 protein level and inhibition of acetylation of histone 3 (AcH3) and histone 4 (AcH4). Our data demonstrate that a certain dose of LIPUS epigenetically regulates adipogenic differentiation.

## Materials and methods

### Isolation and culture of rat visceral preadipocytes

Rat visceral preadipocytes were isolated and cultured as described previously [23]. In our experiment, digestive cell suspension was centrifuged thrice and filtrated twice different from single centrifugation and filtration of conventional method. After filtration using 100 μm mesh, centrifugation will not only be able to remove fibre and impurities, but also separate adipocytes from suspension at 200 × g for 5 min. Then the second centrifugation was at 800 × g for 5 min. This rotating speed could result in cell membrane breakage of adipocytes and remove remaining adipocytes and reduce the loss of preadipocytes in the operation process. Finally, the filtration of 25 μm mesh and the centrifugation can eliminate miscellaneous endothelial cells and isolated preadipocytes were purified in maximum. These cells were cultured in DMEM/F12 medium (Gibco, NY, USA) containing 10% fetal bovine serum (FBS; Hyclone, UT, USA), 100 U/mL penicillin and 100 μg/mL streptomycin (Thermo Scientific, IL, USA) in a 37°C and 5% CO2 humidified incubator. Derived preadipocytes were taken for the identification of cellular immunophenotype by flow cytometry and the number of cells was counted before being seeded in each dish (Figure S2). The culture media was changed every 2 d, and when the cells grow to about 90% fusion, cell suspension was made and inoculated in 6 cm culture dish. Rosiglitazone maleate (ROZ), a specific agonist of PPARγ, GW9662, a PPARγ antagonist [24] and vorinostat (SAHA), a inhibitor of HDACs, were purchased from Selleck.

### Adipocyte differentiation

The procedure of adipocyte differentiation is described below: ① Cells (5 × 105 cells/well) are seeded in 6 cm dishes and are 70–80% confluent after 2 d of culture. Then, cells were maintained to reach contact inhibition for 2 days (Cells are seeded in dishes and are exposed to the daily LIPUS for 10 min from the next day. The LIPUS treatment will last for 4 d and be stopped after adding adipogenic induction medium); ② Remove the DMEM/F12 medium containing 10% FBS and add DMEM/F12 medium containing 5% FBS and differentiation inducers including 1 μM dexamethasone, 1 mM IBMX and 10 μM insulin; ③ Culture cells in adipogenic induction media for 4 d and change the media every 2 d;④ After 4-d incubation, remove the media and add adipogenic maintenance media containing 5% FBS and 10 μM insulin for an additional 4 d. Change the media every 2 d.

### Oil Red-O staining

Cells were fixed in 4% paraformaldehyde for 30 min at room temperature and stained with Oil Red-O dye at room temperature for 10 min. Then cells were rinsed with PBS three times to remove residual Oil Red-O dye and photos were taken under the reverse phase microscope. Black and white pictures showed pre-dyeing cells in the stage of terminal differentiation (4×) and colour pictures showed oil red O-stained cells in the stage of terminal differentiation (20×). Six visual fields were randomly selected under the light microscope in each group and the area of all cells was regarded as total area under each field. The lipid droplet formation was quantitatively analyzed by the ratio of red area to total area. The areas were measured and quantified by using the Image J software.

### Low-intensity pulsed ultrasound stimulation

LIPUS irradiation was performed using a set of ultrasound devices (Agilent Technologies, Santa Clara, CA, USA) which was described previously [23]. We performed LIPUS irradiation under the following conditions: planar transducer frequency 0.5 MHz, voltage applied to each transducer was 400–600 mVpp, pulse repetition frequency 25 Hz, the number of cycles 10, and spatial-temporal average sound pressure 0.8–1.2 MPa. The cell suspension was exposed to the daily LIPUS for 10 min, while the control cell suspension was treated identically except for the absence of the LIPUS stimuli. The temperature of the culture medium in the dishes hardly changed during the ultrasound procedures.

### Flow cytometric analysis of cell proliferation

The EdU (5-ethynyl-2′ -deoxyuridine) is a nucleoside analogue of thymidine that is incorporated into DNA only during DNA synthesis. The preadipocyte proliferation was analyzed by Cell-Light™ EdU Apollo®488 in vitro flow cytometry kit (Ribobio, Guangzhou, China). The Kit provides a method for marking cells in S phase and analyzing DNA replication in proliferating cells. Then, newly synthesized DNA is measured using the 488 nm laser of the flow cytometer according to the manufacturer’s instructions. The scheme for this experiment is described below: ① Cells are seeded in 6 cm dishes and are exposed to the daily LIPUS for 10 min from the next day in treated group; ② The LIPUS treatment will last for 4 d, and then cells were incubated in medium containing EDU (50 μM) for 2 h before collection; ③ The collected cells were incubated in 4% formaldehyde for 15 min at room temperature, and then were washed with PBS; ④ Add 1 mL of 0.5% Triton X-100 in PBS to each tube, then incubate at room temperature for 20 min. Remove the 0.5% Triton X solution; ⑤ Add 0.5 mL of staining reaction solution to each tube, then incubate at room temperature for 10 min, protected from light; ⑥ Remove the reaction solution, then wash each tube with 1 mL of 0.5% Triton X-100 in PBS; ⑦ Cells were resuspended in PBS and analyzed by flow cytometry.

### Western blot

The total proteins were extracted from preadipocytes using standard methods as described previously [23]. Protein samples were separated by SDS-PAGE (6–12%) and transferred onto PVDF membranes, and blocked with bovine serum albumin (BSA). Membranes were incubated with the specific antibodies against p38 (1:1000; Cat. 8690, Cell Signaling Technology, Danvers, MA, USA), p-p38 (1:1000; Cat. 4511, Cell Signaling Technology), ERK (1:1000; Cat. 4695, Cell Signaling Technology), p-ERK (1:1000; Cat. 4370, Cell Signaling Technology), Janus kinase (JNK, 1:1000; Cat. 9252, Cell Signaling Technology), p-JNK (1:1000; Cat. 4668, Cell Signaling Technology), PPARγ (1:500; Cat. 209,350, Abcam, Cambridge, MA, USA), C/EBPα (1:1000; Cat. 2295, Cell Signaling Technology), Lamin B (1:1000; Cat. 13,435, Cell Signaling Technology), GAPDH (1:1000; Cat. 5174, Cell Signaling Technology), HDAC1 (1:1000; Cat. 34,589, Cell Signaling Technology), AcH3 (1:1000; Cat. 8173, Cell Signaling Technology), and AcH4 (1:1000; Cat.2605373, Temecula) at 4°C overnight and then incubated with the appropriate secondary antibody, either peroxidase-labeled anti-rabbit or anti-mouse immunoglobulin, at room temperature. The target proteins were detected using an enhanced chemiluminescence (ECL) kit. Bands were normalized with GAPDH, and protein levels were quantified by Image J.

### Nuclear protein extraction

The nuclear proteins were extracted from preadipocytes using NucBuster Protein Extraction Kit (Millipore, USA) and the procedure of extraction is described below: ①Prepare a single cell suspension using a standard technique; ②Count the cells and transfer a 1.5 ml-tube and centrifuge at low speed (500 × g, 4℃). Remove the supernatant and measure the standard-packed cell volume. 1–3 × 10^7^ cells from a packed cell volume of 50 μL. Up to 250 μL packed cell volume can be processed in a single 1.5 ml-tube; ③Resuspend the cell pellet using 150 μL NucBuster Reagent 1 per 50 μL packed cell volume. Adjust extraction reagent volumes proportionately according to the size of the packed cell volume; ④Vortex 15 sec at high speed. Incubated on ice 5 min, and vortex again 15 sec at high speed; ⑤Centrifuge at 16,000 × g for 5 min 4℃; ⑥Remove the supernatant. A wash with 500 μL of ice-cold PBS may be used at this step to remove additional cytoplasmic proteins; ⑦Resuspend the pellet in 1 μL of resuspended 100×Protease Inhibitor Cocktail, 1 μL of 100 mM DTT, and 75 μL NucBuster Extraction Reagent 2 per 50 μL packed cell volume; ⑧Vortex 15 sec at high speed, incubate on ice 5 min, and again vortex 15 sec at high speed; ⑨Centrifuge at 16,000 × g for 5 min 4℃; ⑩Transfer the supernatant (nuclear extract) to a separate tube. The extract can be used immediately or stored in aliquots for extended periods at −80℃.

### qRT-PCR

Total RNA was isolated and transcribed into cDNA as previously described [23]. The synthesized cDNA was used as template for the analysis of mRNA levels by the SYBR-Green Supermix Kit (Bio-Rad, Hercules, CA, USA) according to the manufacturer’s standard protocol. Equal amounts of cDNA were applied in each reaction mixture. As a control for the specificity of the quantitative real-time PCR, a sample without template was included. The real-time cycler conditions were as follows: initial activation step at 95°C for 10 min, 40 cycles of denaturing at 95°C for 15 s, and annealing/extension at 60°C for 60 s, followed by a melting curve analysis of 65–95°C with 0 .5°C increment, 5 s per step. The following specific primers for target genes were obtained from Invitrogen (Carlsbad, CA, USA): PPARγ, forward 5′-TGACCACTCCCATTCCTTTG-3′, reverse 5′-CAACCATTGGGTCAGCTCTT-3′; C/EBPα, forward 5′-CCATCCGCCTTGTGTGTACT-3′, reverse 5′-GTTTAGCATAGACGCGCACA-3′; C/EBPβ, forward 5′-CGGGTTTCGGGACTTGAT-3′, reverse 5′-CCCGCAGGAACATCTTTAAGT-3′; and C/EBPδ, forward 5′-ATCGACTTCAGCGCCTACAT-3′, reverse 5′-CCGCTTTGTGATTGCTGTT-3′; FABP4, forward 5′-TCATCAGCGTAGAAGGGGACT-3′, reverse 5′-CACGCCCAGTTTGAAGGAA-3′; adiponectin, forward 5′-CGGAGAAGCCGCTTACAT-3′, reverse 5′-CCAGTGCTGCCGTCATAA-3′.

### Identification surface markers of preadipocytes

The surface makers of preadipocytes were identified by flow cytometry and the procedure of identification is described below: ① After 24 h, remove the medium completely, wash the 6-cm dish twice with PBS, and harvest cells with of 0.25% trypsin-EDTA for 5 min at 37°C; ② Neutralize trypsin-EDTA with equal amounts of culture medium, collect cells in a new 15-ml conical tube, and then centrifuge at 400 × g for 7 min.; ③ Decant the supernatant. Wash the pellet twice in ice-cold FCS-wash buffer (PBS containing 10% FBS) using re-suspension by pipetting coupled with centrifugation at 400 × g for 7 min.; ④ Resuspend, count, and adjust preadipocytes to 10^6^ cells/ml in ice-cold FCS-wash buffer. Place cells (100 μl/each tube) into multiple new 15-ml conical tubes and incubate tubes containing preadipocytes at 4°C for 30 min with antibodies against CD29, CD31, CD45 and CD90 (eBioscience, USA) for direct staining. Stop the reaction by washing the cells twice in 10 ml FCS-wash buffer coupled with 400 × g for 7 min; ⑤ Fix and resuspend cells in fixation buffer for flow cytometry.

### RNA interference

Rat small-interfering RNAs against HDAC1 (si-HDAC1) and a scrambled sequence control (si-con) were purchased from Ribobio (Guangzhou, China). To knockdown HDAC1 expression, cells were transfected with si-HDAC1 or si-con for 8 h using Lipofectamine 2000 (Invitrogen, USA) and then were treated with or without LIPUS stimulus.

### The flowchart of experiment

**Figure:**
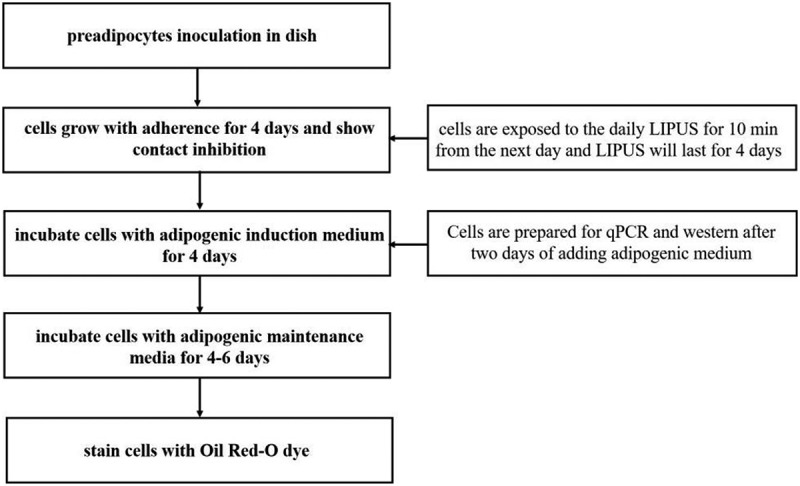

### Statistical analysis

Statistical analyses were performed using GraphPad PRISM 6.01 statistical software (San Diego, California, USA). Data were analyzed using the independent Student’s t-test and were expressed as the mean± SEM of three or more independent experiments (Supplementary material). A p-value <0.05 was considered significant.

## Results

### LIPUS suppresses adipogenic differentiation of visceral preadipocytes

**Figure 1.**
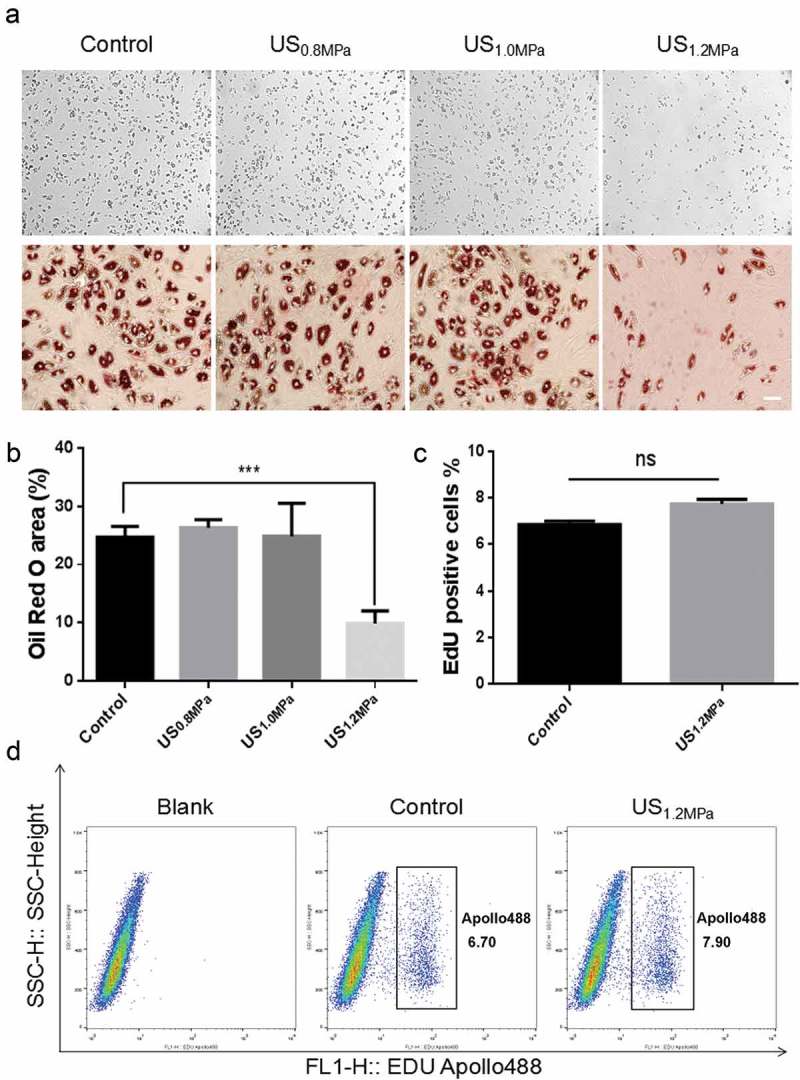
Effects of LIPUS on rat preadipocyte differentiation and proliferation. Primary-cultured preadipocytes were treated with different doses of ultrasound (0.8, 1.0 and 1.2 MPa) for 10 min. (a) Preadipocyte differentiation after different dose of LIPUS treatment was visualized by the oil red O staining. (b) Quantification of lipid droplet formation after different dose of LIPUS treatment. (c,d) Cell proliferation ability was analyzed by flow cytometric analysis using cell-light EdU Apollo 488 kit staining. All values are expressed as the mean ± SEM of three independent trials. Data were analyzed with independent *t* test. *** p < 0.001. Bar: 50 μm.

Previous studies have demonstrated that the early apoptosis of rat visceral preadipocytes is obviously promoted by LIPUS stimulation[24]. However, the influence of an external acoustic stress on adipogenic differentiation of preadipocytes has never been clearly elucidated. To explore the effects of LIPUS on adipocytes differentiation, we observed the adipogenic differentiation of rat visceral preadipocytes under different dose of daily LIPUS stimulation (Supplementary Table). Adipogenic differentiation was estimated by the formation of lipid droplets in preadipocytes visualized by oil red O staining. The results showed that the *in situ* peak negative pressure of the source transducer 1.2 MPa led to a prominent inhibition of lipid droplets formation compared to untreated cells (Figure 1(a,b)). To exclude that LIPUS increased cell proliferation prior to adipogenic differentiation [25], we examined the effect of LIPUS on preadipocyte proliferation. Proliferation assay by EdU staining showed that the number of EdU positive cells was not influenced after LIPUS treatment (Figure 1(c,d)). These data suggested that LIPUS could suppress adipogenic differentiation of preadipocytes without affecting cell proliferation.

### LIPUS influences C/EBPα and PPARγ pathway in transcription and translation level

**Figure 2.**
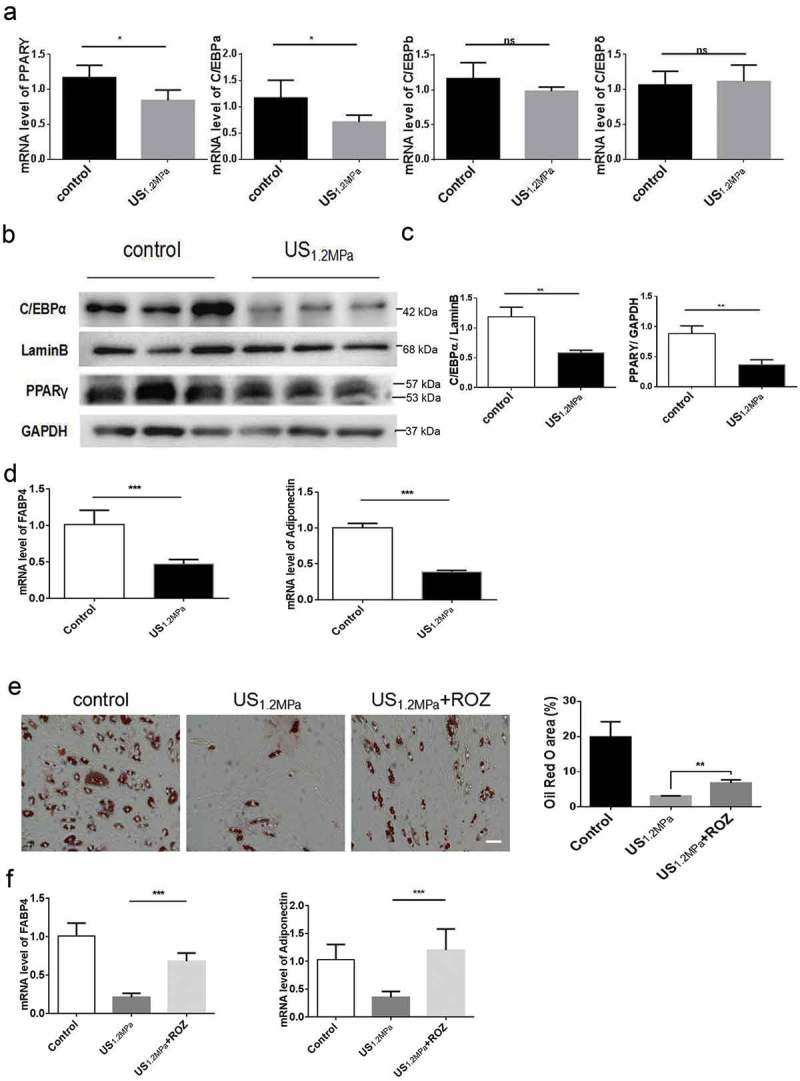
Effects of LIPUS on gene expression associated with adipogenic differentiation. Cells were prepared for qPCR and western after 2 d of adding adipogenic medium. We chose this time point because the levels of C/EBPs and PPARγ will be obviously up-regulated in the stage of initial differentiation and the levels of C/EBP beta and delta will not change in the stage of terminal differentiation compared to the stage of preadipocyte. (a) The mRNA levels of PPARγ, C/EBPα, C/EBPβ and C/EBPδ were examined by qRT-PCR assay in differentiated preadipocytes after LIPUS treatment. (b,c) The protein expression levels of PPARγ and C/EBPα were assessed by western blotting in differentiated preadipocytes after LIPUS treatment. (d) The mRNA levels of representative adipogenic markers including Fabp4 and adiponectin after LIPUS treatment were examined by qRT-PCR. (e) Preadipocytes were treated with the PPARγ agonist ROZ (1 μM). The effects of ROZ on the LIPUS-induced inhibition of preadipocyte differentiation were measured by oil red O staining. (f) The effects of ROZ on adipogenic differentiation markers after LIPUS treatment were examined by qRT-PCR. All values are expressed as the mean ± SEM of three independent trials. Data were analyzed with independent *t* test. * *p*< 0.05, ** *p*< 0.01, *** *p*< 0.001. Bar: 50 μm.

PPARγ and the three members of the C/EBPs family of transcription factors play critical roles during adipogenic differentiation [8]. To clarify the molecular basis of LIPUS on adipogenic differentiation of preadipocytes, we measured the expression of PPARγ, C/EBPα, C/EBPβ and C/EBPδ. We found that LIPUS treatment downregulated the PPARγ and C/EBPα mRNA levels but did not affect the C/EBPβ and C/EBPδ mRNA levels (Figure 2(a)). Consistent with the changes of mRNAs, western blot analysis showed that LIPUS significantly reduced the protein levels of PPARγ and C/EBPα compared to the control group (Figure 2(b,c)). In accordance with the results of lipid droplets staining, mRNA expression levels of the adipogenic marker genes Fabp4 and adiponectin were significantly decreased by LIPUS treatment (Figure 2(d)). To further evaluate the effect of PPARγ in regulating the LIPUS-induced suppression of adipogenic differentiation, preadipocytes were treated with the PPARγ agonist ROZ. The activation of PPARγ partially rescued the decreased lipid droplets formation (Figure 2(e)) and the decreased expression of adipogenic differentiation markers (Figure 2(f)).

### LIPUS up-regulates HDAC1 expression and affects histone 3 and histone 4 acetylation modification

**Figure 3.**
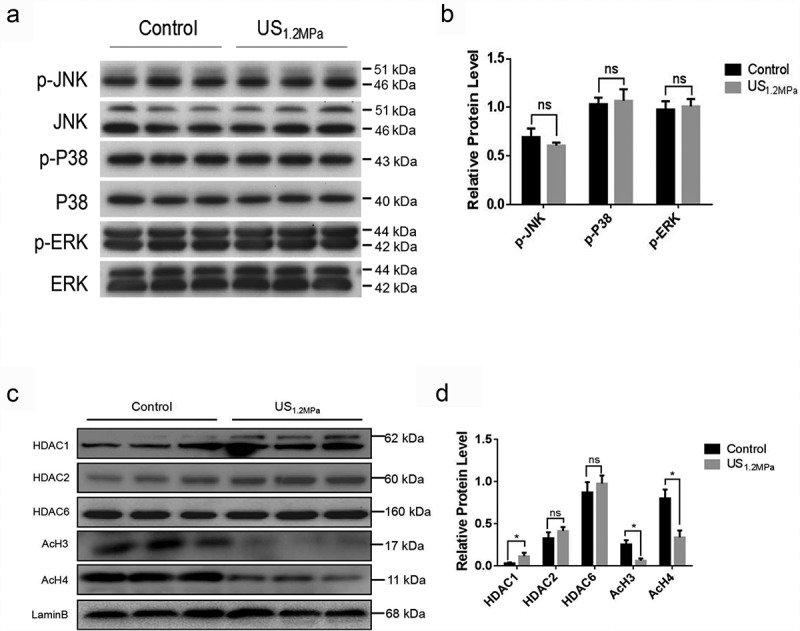
LIPUS increases HDAC1 expression and decreases histone 3 and histone 4 acetylation modification. (a) The phosphorylation levels of ERK, P38 and JNK were analyzed by western blotting. (b) Quantification of p-ERK, p-P38 and p-JNK normalized to their total proteins. (c) Nuclear protein levels of HDAC1, HDAC2, HDAC6, AcH3 and AcH4 were assessed by western blotting. (d) Quantification of HDAC1, HDAC2, HDAC6, AcH3 and AcH4 normalized to Lamin B. Relative protein levels were analyzed with independent *t* test. All values are expressed as the mean ± SEM of three independent trials. * P < 0.05.

Previous reports have showed that ERK phosphorylation is crucial for the inhibitory effects of adipogenesis and stimulation with external acoustic stress could suppress 3T3-L1 cell differentiation through ERK signalling pathway [26]. Thus, we examined the activation of ERK, P38 and JNK by western blotting. In contrast to the effect of LIPUS in 3T3-L1 cells, LIPUS treatment did not affect the protein levels of p-ERK, p-P38 and p-JNK in differentiated rat visceral adipocytes (Figure 3(a,b)). HDAC1 have been reported to be involved in regulating C/EBPα transcription and inhibiting preadipocyte differentiation [12,27]. We then tested whether the attenuated differentiation of preadipocytes triggered by LIPUS was associated with the changes of HDAC1 level. As illustrated in Figure 3(c,d), LIPUS markedly increased nuclear protein level of HDAC1 and did not affect the levels of HDAC2 and HDAC6 compared with the control group. Inconsistent with the effect of HDAC1, obviously decreased levels of AcH3 and AcH4 were observed in the LIPUS-treated group.

### SAHA could rescue the effects of LIPUS on adipogenic differentiation

**Figure 4.**
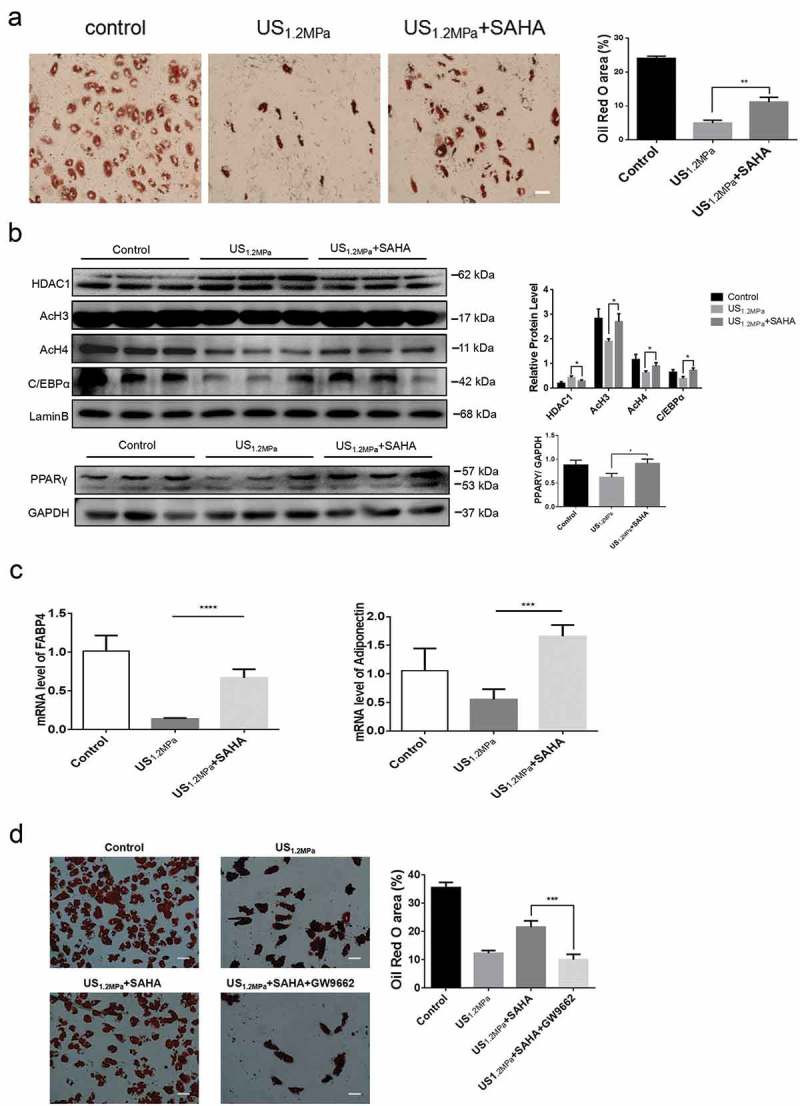
SAHA could rescue the effects of LIPUS on adipogenic differentiation. Preadipocytes were treated with LIPUS combined with the HDACs inhibitor SAHA. (a) The effects of HDAC1 inhibition on preadipocyte differentiation after LIPUS treatment were evaluated by the oil red O staining. (b) The effects of HDAC1 inhibition on protein levels of PPARγ, C/EBPα, HDAC1, AcH3 and AcH4 after LIPUS treatment were assessed by western blotting. Quantification of indicated proteins normalized to lamin B(HDAC1, C/EBPα, AcH3 and AcH4) or GAPDH(PPARγ). (c) The effects of HDAC1 inhibition on the mRNA expression of adipogenic differentiation markers after LIPUS treatment were examined by qRT-PCR assay. (d) The effect of GW9662 on the SAHA’s effect after LIPUS treatment were assessed by the oil red O staining. All values are expressed as the mean ± SEM of three independent trials. Data were analyzed with independent *t* test. * *p*< 0.05, ** *p*< 0.01, *** *p*< 0.001, **** *p*< 0.0001. Bar: 50 μm.

Preadipocytes were treated with both LIPUS and SAHA regarded as HDACs inhibitor. The SAHA treatment rescued the LIPUS-induced reduction of lipid droplets formation (Figure 4(a)). Western blotting assay showed that SAHA decreased the level of HDAC1 and blocked the decreased levels of C/EBPα, PPARγ, AcH3 and AcH4 induced by LIPUS (Figure 4(b)). Meanwhile, the SAHA rescued the decreased mRNA levels of adipogenic differentiation markers (Figure 4(c)). In addition, we made use of PPARγ antagonist GW9662 (5 μM) to illuminate the effect of PPARγ antagonism on the SAHA’s effect. As shown in Figure 4(d), PPARγ antagonist could reverse the recovery effect of SAHA on the inhibition of adipogenic differentiation induced by LIPUS.

### Down-regulation of HDAC1 by SiRNA reverses the effects of LIPUS on adipogenic differentiation

**Figure 5.**
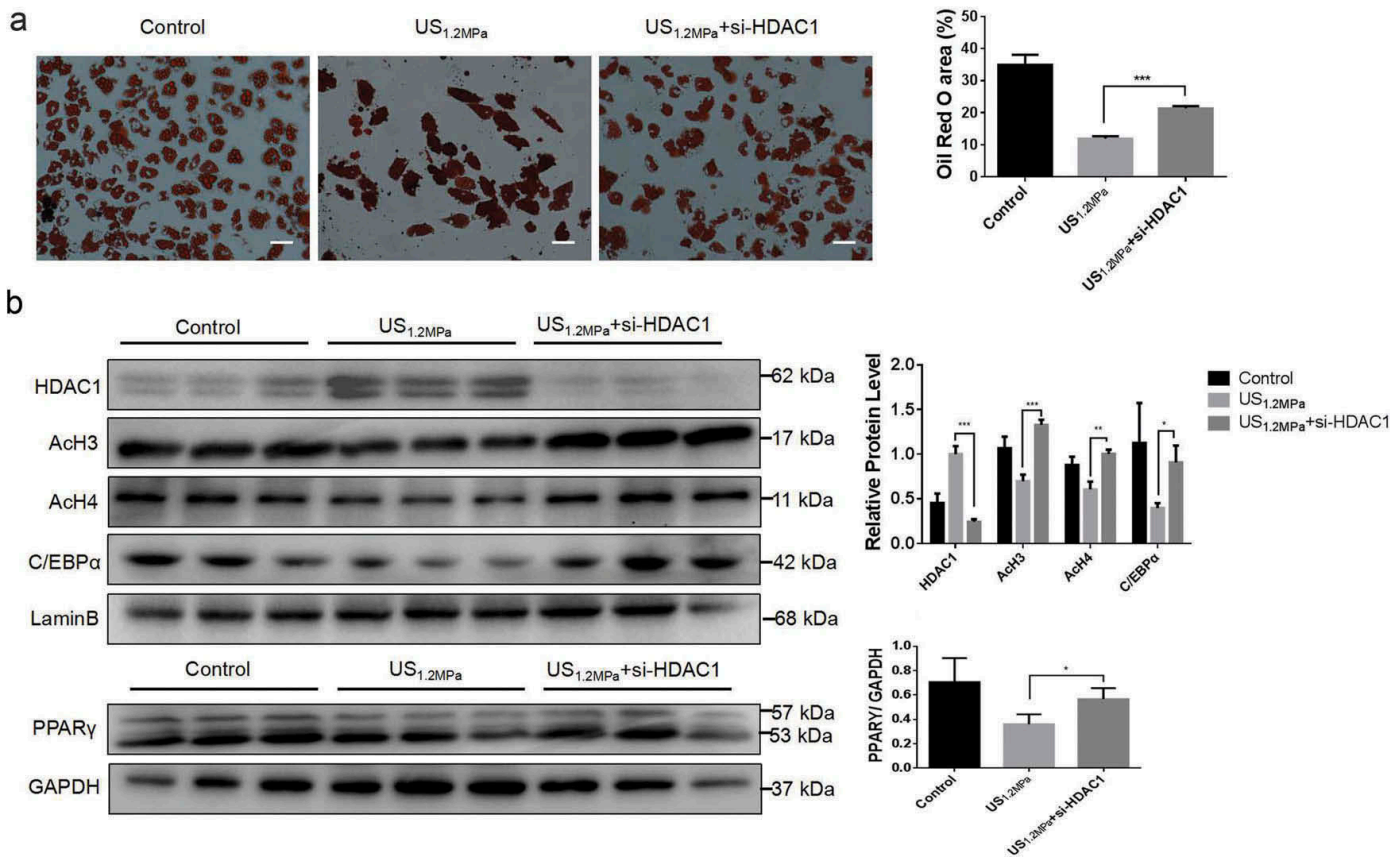
Down-regulation of HDAC1 by siRNA reverses the effects of LIPUS on adipogenic differentiation. Preadipocytes were treated with LIPUS combined with the si-HDAC1. (a) The effects of HDAC1 inhibition by si-RNA on preadipocyte differentiation after LIPUS treatment were evaluated by the oil red O staining. (b) The effects of HDAC1 inhibition by si-RNA on protein levels of PPARγ, C/EBPα, HDAC1, AcH3 and AcH4 after LIPUS treatment were assessed by western blotting. Quantification of indicated proteins normalized to lamin B(HDAC1, C/EBPα, AcH3 and AcH4) or GAPDH(PPARγ). All values are expressed as the mean ± SEM of three independent trials. Data were analyzed with independent *t* test. * *p*< 0.05, ** *p*< 0.01, *** *p*< 0.001. Bar: 50 μm.

The SAHA treatment could partially reverse the inhibitory effect of LIPUS on adipocyte differentiation. SAHA is a general HDACs inhibitor and it didn’t mean that the effect of SAHA was mediated by HDAC1. Therefore, HDAC1 was specifically knocked down by siRNA. As showed in results, the specifical down-regulation of HDAC1 could rescue the reduction of lipid droplets formation (Figure 5(a)) and the decrease of C/EBPα, PPARγ, AcH3 and AcH4 induced by LIPUS (Figure 5(b)).

## Discussion

Compared with traditional treatment options, LIPUS has advantage of having good security and avoiding accumulative injury to the surrounding normal tissues. LIPUS is a kind of micromechanical stress that is already clinically applied in treating several diseases including tumours and bone fracture healing. Numerous studies have also revealed the bio-effects of LIPUS such as increasing cell membrane penetrability and regulating cell apoptosis, proliferation, differentiation and migration [28–32]. Our previous study has found that an average intensity of 109.44 mW/cm^2^ significantly promoted rat visceral preadipocyte apoptosis via elevating p-p38 [23]. In order to reduce the thermal effect of ultrasound as much as possible, we decrease the number of cycles and pulse repetition frequency, and regulate voltage applied to each transducer during the course of the experiment. These results suggested that LIPUS may be used for regulating adipocyte differentiation in visceral adipose tissues, then providing a valuable strategy for alleviating visceral obesity and related diseases.

It is known that adipogenic differentiation is regulated by some key adipogenic transcription factors [33]. PPARγ is recognized as a master transcriptional factor of adipogenesis for preadipocyte differentiation. Three members of the C/EBPs(C/EBPα, β and δ) family of transcription factors have been regarded as serving a critical role in adipogenesis in association with PPARγ. In addition, Fabp4 and adiponectin, the representative adipogenic markers, are also involved in the process of lipid droplet accumulation [34]. Our data showed that LIPUS obviously decreased the mRNA levels of C/EBPα, PPARγ, Fabp4 and adiponectin during adipogenic differentiation of rat visceral preadipocytes, and activation of PPARγ by ROZ partially reversed the effect of LIPUS on adipogenesis. These data suggest that LIPUS mediated suppression of adipogenesis is dependent on the downregulation of PPARγ and C/EBPα.

The ERK activation is a crucial signal event regulating the differentiation of various cell types, including osteoblasts and adipocytes [35]. Some researchers found that a 30 mW/cm^2^ dose of LIPUS could increase ERK phosphorylation and influence the multilineage differentiation of mesenchymal stem and progenitor cell lines [26]. Furthermore, one study also proved that apelin suppresses adipogenic differentiation through an ERK-dependent pathway in preadipocytes and mature adipocytes [36]. We also study the association between the level of ERK activation and LIPUS-induced inhibition of adipogenic differentiation in rat visceral preadipocytes. However, a 1.2 MPa dose of LIPUS treatment did not appear to affect protein levels of p-ERK, p-P38 and p-JNK in this study. Since our dose of LIPUS is much lower than reported dose from other studies, we think that ERK is not the only factor involved in LIPUS mediated suppression of adipogenesis. Our study also suggest that the low dose with high acoustic pressure may be a good strategy for therapeutic LIPUS.

HDACs have been reported to be involved in the suppression of transcriptional responses and to be involved in crucial cellular process such as differentiation of the 3T3-L1 cells by suppressing transcriptional activity of key genes, including C/EBPα [13,37]. Some studies have demonstrated that HDAC1 acts as an inhibitor of the adipogenic process via abrogating C/EBPα and PPARγ transcription [35]. However, no data has been reported linking the effect of LIPUS with nuclear HDAC1 levels until recently. In the current study, we found that LIPUS could inhibit preadipocyte differentiation accompanied by the up-regulation of HDAC1 and down-regulation of AcH3 and AcH4. Inhibition of HDAC1 by SAHA rescued the LIPUS mediated suppression of adipogenic differentiation and the reduction of PPARγ, C/EBPα and adipogenic markers. Our data suggest that LIPUS inhibits preadipocyte differentiation in a HDAC1-dependent manner. LIPUS may be used as a HDAC1 activator to alleviate some adverse effects in normal tissues caused by clinical application of HDAC inhibitor in cancer therapy.

Because of the different purposes of experiments, we used a different mode of LIPUS in this study. In our previous study, the higher dose of LIPUS was used to investigate its pro-apoptotic effects on preadipocytes. However, in this study, we aimed to reveal the mode which can affect adipocyte differentiation without promoting apoptosis. So preadipocytes were treated with lower dose and longer time of LIPUS. The mode of LIPUS could inhibit adipocyte differentiation and down-regulate the level of PPARγ which is regarded as the key transcriptional regulator of adipocyte differentiation. Then the levels of p-P38 and PPARγ were not up-regulated because the mode did not affect cell apoptosis. Our data demonstrated that LIPUS performs various biological functions in different parameter conditions.

Taken together, our study demonstrated that micromechanical stimulus by LIPUS suppresses primary visceral preadipocyte differentiation. The LIPUS-induced effects are mediated by HDAC1 and PPARγ signalling. Our study suggests a potential strategy for clinical obesity, especially visceral obesity management via LIPUS.
