# Supplementary figures and images for: Low-intensity pulsed ultrasound inhibits adipogenic differentiation via HDAC1 signalling in rat visceral preadipocytes
Source: Adipocyte. 2019 Jul 19;8(1):292–303. doi: 10.1080/21623945.2019.1643188 (PMC6768184; doi:10.1080/21623945.2019.1643188)

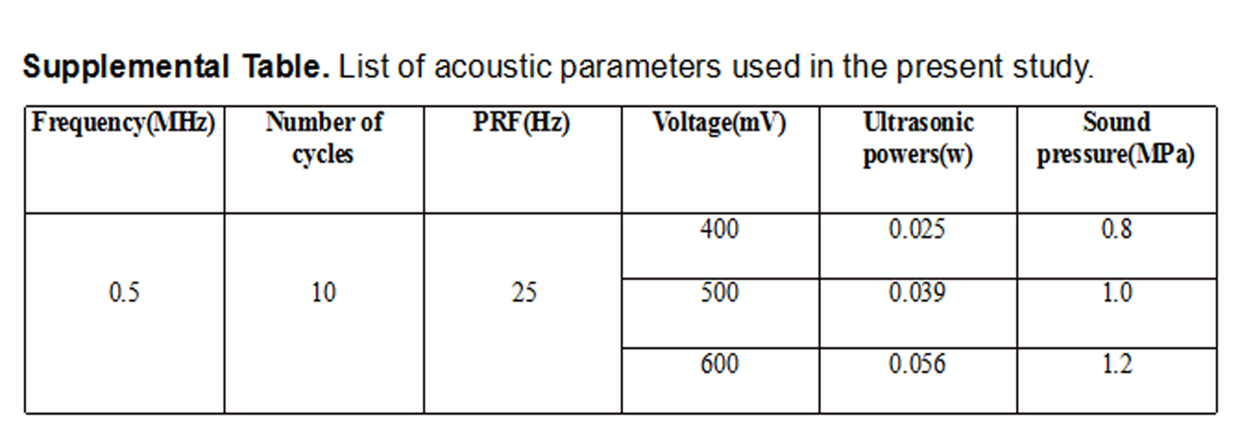

Supplement: Supplemental Material [file kadi-08-01-1643188-s001.zip › Supplemental table.tif]

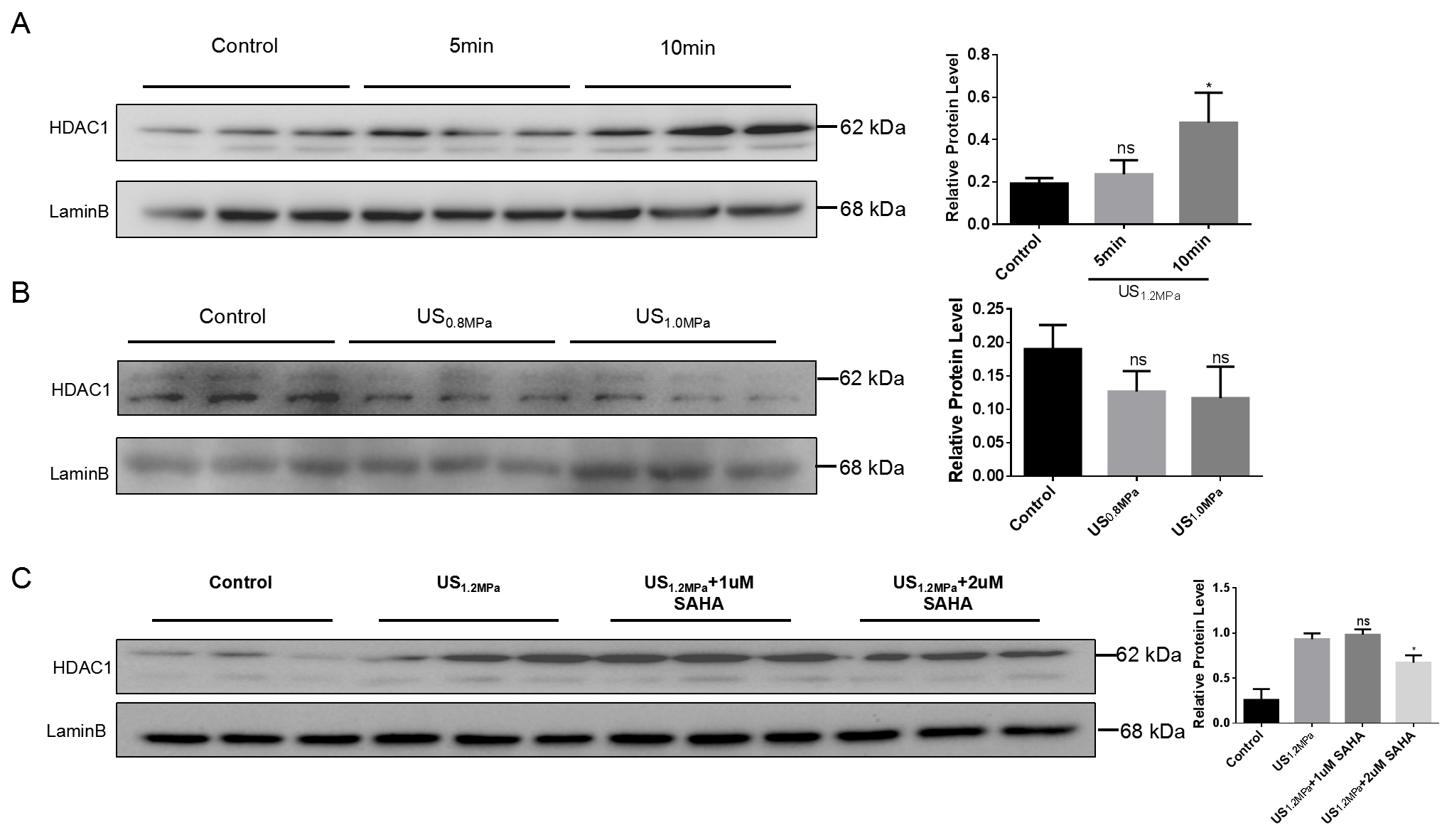

Supplement: Supplemental Material [file kadi-08-01-1643188-s001.zip › Supplementary figure 1.tif]

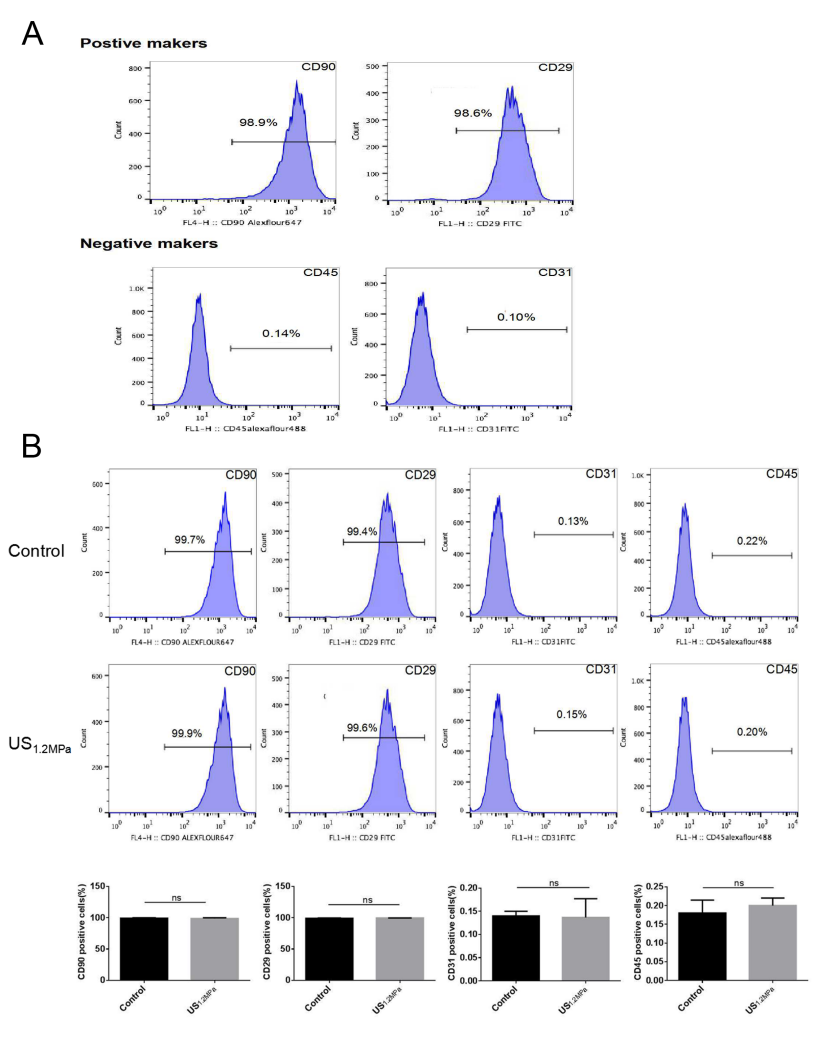

Supplement: Supplemental Material [file kadi-08-01-1643188-s001.zip › Supplementary figure 2.tif]

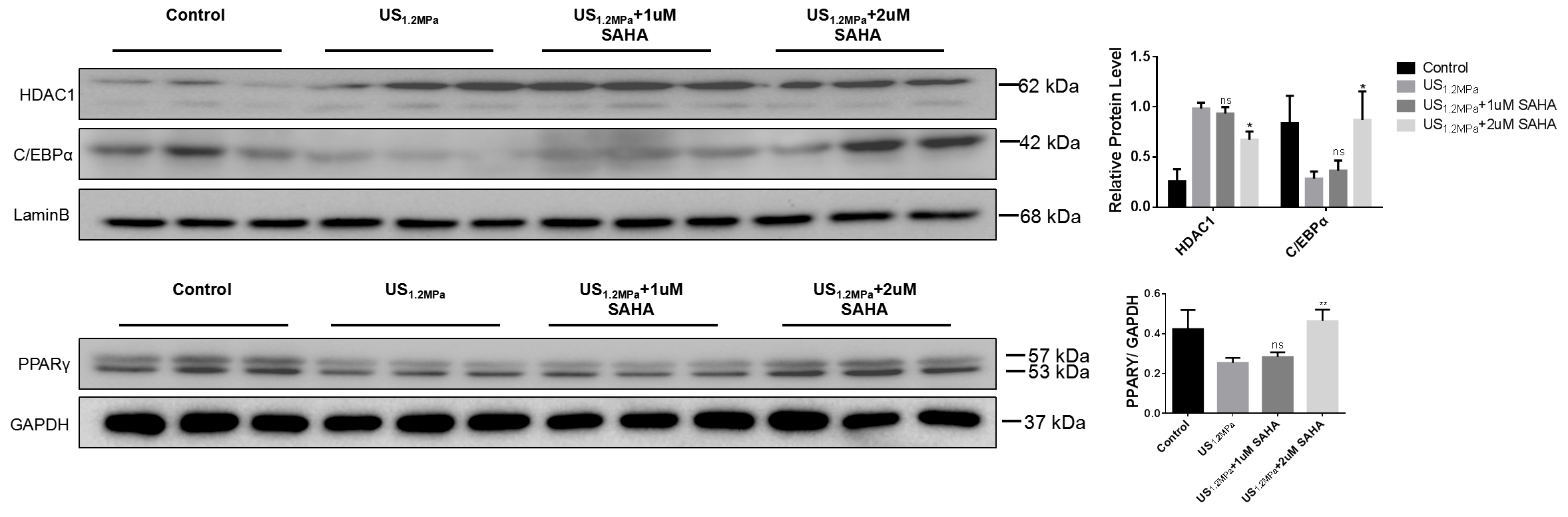

Supplement: Supplemental Material [file kadi-08-01-1643188-s001.zip › Supplementary figure 3.tif]
